# Analysis of Shear Bond Strength and Morphology of Er:YAG Laser-Recycled Ceramic Orthodontic Brackets

**DOI:** 10.1155/2016/7276287

**Published:** 2016-03-07

**Authors:** Ruo-qiao Han, Kai Yang, Ling-fei Ji, Chen Ling

**Affiliations:** ^1^Department of Orthodontics, Capital Medical University School of Stomatology, Dongcheng District, Beijing 100050, China; ^2^Institute of Laser Engineering, Beijing University of Technology, Chaoyang District, Beijing 100124, China

## Abstract

*Objective*. The aim of this study was to compare the recycling of deboned ceramic brackets via an Er:YAG laser or via the traditional chairside processing methods of flaming and sandblasting; shear bond strength and morphological changes were evaluated in recycled brackets versus new brackets.* Materials and Methods*. 3M Clarity Self-Ligating Ceramic Brackets with a microcrystalline base were divided into groups subjected to flaming, sandblasting, or exposure to an Er:YAG laser. New ceramic brackets served as a control group. Shear bond strengths were determined with an Electroforce test machine and tested for statistical significance through analysis of variance. Morphological examinations of the recycled ceramic bracket bases were conducted with scanning electron microscopy and confocal laser scanning microscopy. Residue on the bracket base was analyzed with Raman spectroscopy.* Results*. Faded, dark adhesive was left on recycled bracket bases processed via flaming. Adhesive was thoroughly removed by both sandblasting and exposure to an Er:YAG laser. Compared with new brackets, shear bond strength was lower after sandblasting (*p* < 0.05), but not after exposure to an Er:YAG laser. The Er:YAG laser caused no damage to the bracket.* Conclusion*. Er:YAG lasers effectively remove adhesive from the bases of ceramic brackets without damaging them; thus, this method may be preferred over other recycling methods.

## 1. Introduction

Lasers, which are increasingly employed in oral medicine, have good monochromaticity, excellent coherence, strong directionality, and high brightness. In orthodontics, lasers are used for debonding brackets [1, 2], accelerating tooth movement [3, 4], managing dislodged brackets [5, 6], and preventing enamel demineralization around brackets [7, 8].

Since their commercialization in 1986 [9], ceramic brackets have been favored by adult patients and orthodontic doctors due to their aesthetics, which are better than those of metal brackets. During fixed orthodontic treatments, the rebonding of brackets for various reasons is difficult to avoid [10]. Replacing a new ceramic bracket each time can be not only a waste of resources but also an economic burden on patients. Since ceramic brackets are expensive and maintain their shape and complete structure [11] after falling off, it is of great significance to develop methods of recycling and rebonding dislodged ceramic brackets in the same patients. Previous findings have varied with regard to the effectiveness of flaming [12, 13], silica coating [14, 15], and sandblasting [16, 17]. In 2013, Ahrari et al. [6] reported that an Er,Cr:YSGG laser removed most of the adhesive from dislodged ceramic bracket bases with some degrees of damage to the ball-base and that the shear bond strength of recycled brackets was comparable to that of new brackets. This investigation prompted us to evaluate whether a laser could be used to facilitate the refurbishing of ceramic brackets.

Er:YAG and Er,Cr:YSGG lasers are emitted in the wavelengths of 2,940 *μ*m and 2,780 *μ*m, respectively, and these wavelengths match two of the absorption peaks of water. Specifically, the wavelength of the Er:YAG laser (2,940 *μ*m) is indicated for the treatment of hard and soft tissues [17], so this kind of laser is applied widely in clinical medicine.

In this study, we measured the shear bond strengths of new brackets and dislodged brackets processing by flaming and ultrasonic cleaning, sandblasting, or exposure to an Er:YAG laser. The adhesive remnant index (ARI) was recorded after removal of the ceramic brackets. Scanning electron microscopy (SEM) was employed to examine the morphology of new bracket bases and processed bases. Confocal laser scanning microscopy (CLSM) was used to observe the three-dimensional structure of bracket bases, and T64000 Raman spectroscopy was used to analyze the ingredients in bracket bases.

## 2. Materials and Methods

A total of 105 premolar teeth extracted for orthodontic reasons were collected from Beijing Stomatological Hospital. This study was approved by the local Ethics Committee (number 2013-06). Periodontal tissue remnants were removed cleanly, and teeth were stored in 0.9% NaCl at 4°C for up to 6 months until use. All teeth were examined under 10x magnification; any carious, damaged, obviously cracked, hypoplastic, or tetracycline-stained teeth or teeth with dental fluorosis were rejected. Forty-five teeth were used to prepare recycled ceramic brackets for three experimental groups, and the other 60 teeth were separated into four groups for analysis of shear bond strength. Clarity Self-Ligating Ceramic Brackets (3M Unitek, USA) with a microcrystalline base were used in this investigation. Sixty maxillary premolar brackets were allocated to the four groups (*n* = 15 brackets per group).

### 2.1. Sample Preparation and Group Design

Forty-five new ceramic brackets were bonded to unetched and slightly wet tooth surfaces with Transbond XT adhesive (3M Unitek, USA), allowing easy separation of the bonded brackets from the teeth with tweezers. Before bonding, the buccal enamel of each tooth was cleaned with nonfluoridated pumice powder and rubber prophylactic cups for 20 s, rinsed, dried with air spray, and etched with a 35% gel of phosphoric acid (Heraeus Kulzer, Germany) for 30 s. Sixty ceramic brackets were bonded to teeth with Transbond XT adhesive in accordance with the manufacturer's instructions. Excess resin around the bracket base was removed with a dental probe. Adhesives were light-cured for 10 s on each side of the bracket with a curing light (Beyond, USA).

A total of 45 recycled ceramic brackets were generated and randomly divided into three experimental groups; the control group consisted of 15 bonded, new ceramic brackets. In the flame group, previously bonded (recycled) ceramic brackets were processed via flaming, which was achieved by heating the bracket base on an alcohol burner and burning off the adhesive, rinsing the base under high-pressure water vapor, ultrasonic cleaning for 5 min, and blow-drying. In the sandblasting group, previously bonded (recycled) ceramic brackets were processed via sandblasting using a MacroCab Danville Engineering sandblasting machine (MacroCab, USA) with 50 *μ*m aluminum oxide abrasive powder (Hager & Werken, Germany), maintaining a 5 mm distance between the ceramic bracket base and the handpiece head, and sandblasting until the adhesive was not visible to the naked eye and then rinsed with water-air spray for 15 seconds to remove residual powder. In the laser group, previously bonded (recycled) ceramic brackets were processed with an Er:YAG laser (Doctor Smile, Italy) at a wavelength of 2940 nm, a beam diameter of 400 *μ*m, an energy density of 60 J/cm^2^, an irradiant power of 6 W, and a repetition rate of 20 Hz, with the ceramic bracket base held perpendicular to the laser until adhesive removal was complete. The operator wore special goggles to protect the eyes during laser exposure. All bonding procedures were performed by the same researcher.

### 2.2. Shear Bond Strength

Specimens were submerged in distilled water at 37°C for 24 h. Shear bond strength was measured with a universal testing machine (AG-X, Shimadzu, Japan). The cutting blade was placed between the bracket's wing and the base, parallel to the base and perpendicular to the slot of the bracket [18]. Debonding was accomplished with a bar speed of 1 mm/min until the bracket dislodged; a computer recorded the maximum force.

### 2.3. ARI

After testing of bond strength, the amount of adhesive residue on each tooth surface was observed under a stereomicroscope at 10x magnification. Enamel surfaces were scored according to ARI: 0, no adhesive left on the tooth; 1, ≤50% of the adhesive left on the tooth; 2, >50% of the adhesive left on the tooth; and 3, all adhesive left on the tooth, with a distinct impression of the bracket base [19].

### 2.4. Morphology of Ceramic Bracket Bases and Residual Component Analysis

Three brackets were randomly selected from each group. The morphology of the ceramic bracket bases before and after processing was observed by spraying carbon under SEM at 300x magnification (SS550, Shimadzu, Japan). CLSM (OLS3100, Olympus, Japan) was used to detect three-dimensional changes in the ceramic bracket base. Component analysis of residues on the base was conducted with Raman spectroscopy (T64000, Horiba Jobin Yvon, France). Spectroscopy data were processed with OriginPro 8 software to produce a Raman spectrogram. Components were evaluated based on the occurrence of the Raman characteristic displacement peak compared with the Raman spectra database.

### 2.5. Statistical Analysis

All statistical analyses were conducted with SPSS 19.0 for Windows (IBM, USA). The Kolmogorov-Smirnov test indicated that the data for shear bond strength were normally distributed; these data were subsequently subjected to analysis of variance. Further comparisons between groups were conducted with the least significant difference test. ARI scores were analyzed using the Kruskal-Wallis test. The threshold for significance was set at *p* < 0.05.

## 3. Results

The shear bond strength of recycled ceramic brackets processed by sandblasting was lower than that of new brackets (*p* = 0.00; Table 1). However, the shear bond strength of neither the flame group and nor the laser group significantly differed from the control group (flame *p* = 0.79, laser *p* = 0.90; Table 1), indicating that flame and laser processing were better than sandblasting for ceramic brackets.

ARI scores did not differ between the experimental and control groups (*p* > 0.05; Table 2). However, the ARI scores for sandblasted brackets were higher than those for other brackets (Table 2), suggesting that more adhesive was left on the surface of these teeth and that lower relative bond strength was achieved between the adhesive and sandblasted brackets.

**Figure 1:**
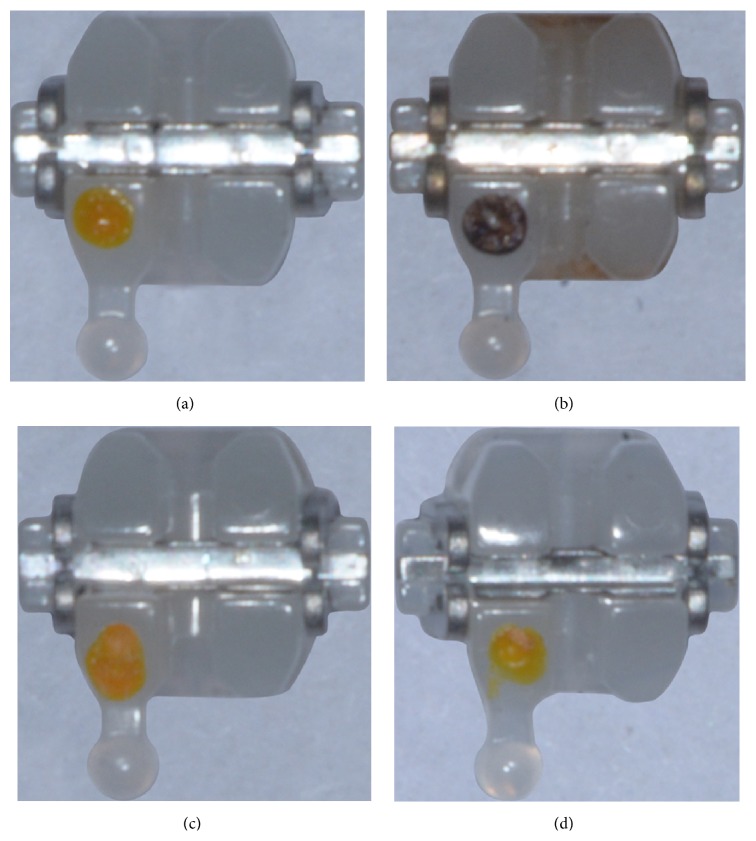
Photographs of ceramic brackets. (a) New bracket. (b) Flame-processed bracket. (c) Sandblasted bracket. (d) Bracket exposed to an Er:YAG laser.

New ceramic brackets had a clear porcelain appearance, while brackets processed by flaming were faded and dark (Figure 1). The colors of brackets exposed to sandblasting or to the laser were similar to that of control brackets (Figure 1).

**Figure 2:**
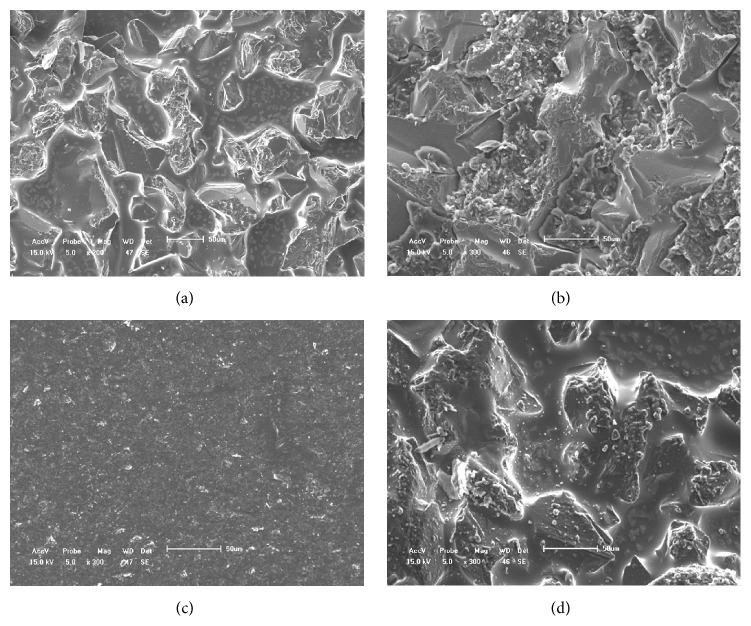
SEM (300x magnification) of the bases of ceramic brackets. (a) New bracket. (b) Flame-processed bracket. (c) Sandblasted bracket. (d) Bracket exposed to an Er:YAG laser.

**Figure 3:**
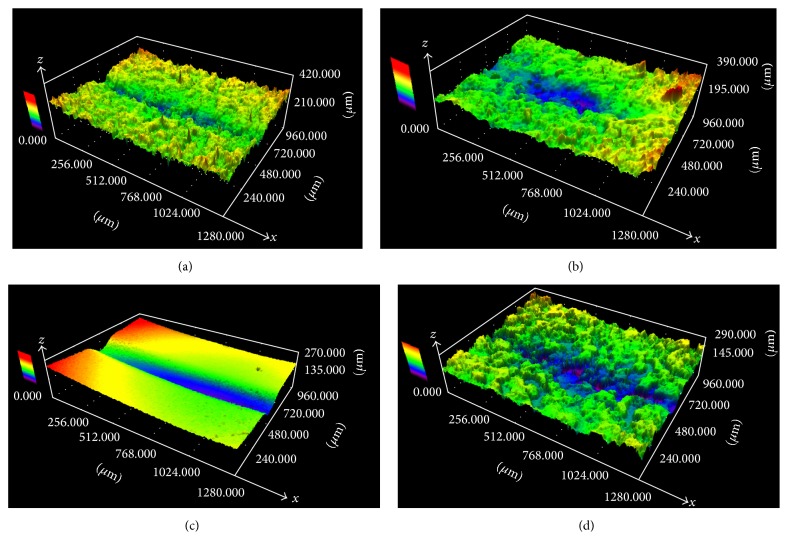
Three-dimensional reconstruction of the bases of ceramic brackets. (a) New bracket. (b) Flame-processed bracket. (c) Sandblasted bracket. (d) Bracket exposed to an Er:YAG laser.

SEM and CLSM demonstrated that new ceramic bracket bases were made up of irregular microcrystalline structures. Some residual adhesive was evident in the hollows of the microcrystalline structures of ceramic brackets processed by flaming (Figure 2). Sandblasting removed all adhesive, but destroyed the bracket bases (Figure 2). However, the Er:YAG laser removed all adhesive completely and maintained the integrity of the microcrystalline structure (Figures 2 and 3).

**Figure 4:**
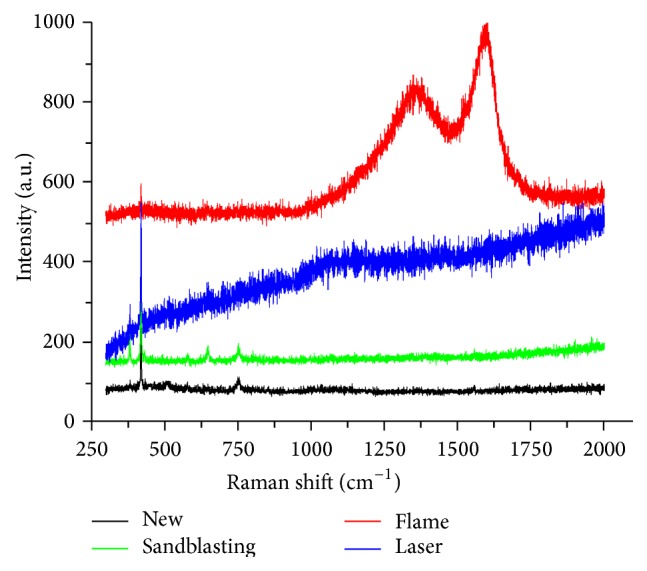
Raman spectroscopy.

An overlapping Raman peak related to aluminum oxide was visible on Raman spectroscopy (Figure 4). For ceramic brackets processed by flaming, the other Raman peak on the spectrogram suggested that carbide remained on the base (the peak reflected the ash of the adhesive; Figure 4). No other Raman peak appeared for new brackets or for brackets processed via sandblasting or the laser (Figure 4), indicating that there was no adhesive on the bases of these brackets. These results of the Raman analysis were consistent with SEM and CLSM.

## 4. Discussion

Flaming is a very old method for removing adhesive from the bases of ceramic brackets. Lew et al. [12] heated used ceramic brackets to burn off the residual composite resin from the bracket base. After cooling the brackets to room temperature, Lew et al. removed residual composite resin by lightly scraping the bracket base. The brackets were then rinsed with 100% alcohol and resilanized with a thin layer of porcelain primer. Lew et al. [12] reported that the shear bond strength of dislodged ceramic brackets processed by the flaming method was significantly lower than that of new brackets. Martina et al. [13] also detected a decrease in shear bond strength when ceramic brackets were processed via flaming and ultrasound. In the current study, flaming reduced the shear bond strength of ceramic brackets relative to new brackets, but the difference was not statistically significant. However, SEM and CLSM revealed residual adhesive in the hollows of the microcrystalline structure of the bracket base. Raman spectroscopy suggested that this residue was carbide or ash from the adhesive. In addition, ceramic brackets processed by flaming were faded and dark. Due to these poor aesthetics, we do not recommend the flaming method for recycling ceramic brackets.

Sandblasting is a surface-roughening method that was originally used to improve the bond strength of new brackets. Sandblasting is commonly used to remove adhesive from the bracket base, which is effective for rebonding recycled metal brackets [20]. Most previous reports indicated that shear bond strength significantly decreased when ceramic brackets processed by sandblasting were rebonded. The microcrystalline structures on the base of ceramic bracket are fine and fragile, so we chose a smaller granule, 50 *μ*m aluminum oxide abrasive powder, to manage the brackets in order to get less destruction of the bases. Here, we found that sandblasting removed the adhesive from the bracket base, but sandblasted brackets displayed significantly less bond strength than new brackets. This effect may be due to destruction of the bracket base during sandblasting. Although sandblasting roughens the surface of the bracket base, the reduction in bonding strength detected here was far less than what could have resulted from damage to the microcrystalline structure. Brackets are retained on the tooth surface via mechanical interaction between the adhesive and the microcrystalline structure of the bracket; thus, damage to the microcrystalline structure directly influences bond strength.

In recent years, as use of lasers in dentistry is increasing, the hospital does not need to buy the sophisticated equipment for recycling the brackets specially. Processing of metal brackets with a laser was previously successful [21, 22]. In 2013, Ahrari et al. [6] concluded that an Er,Cr:YSGG laser removed most of the adhesive from the bases of dislodged ceramic brackets with some degrees of damage to the ball-base, yielding a shear bond strength that was comparable to that of new brackets. In the present study, exposure of ceramic brackets to an Er:YAG laser at a wavelength of 2940 nm led to complete removal of the adhesive. Absorption of light from an Er:YAG laser is considerably greater in water than in air; the laser transfers enough energy to the water to destroy the adhesive on the bracket. In addition, the use of air and water spray during adhesive removal prevents excessive increases in the temperature of the ceramic bracket. We detected no significant differences between new brackets and brackets exposed to an Er:YAG laser, and the microcrystalline structures of laser-exposed brackets were not damaged. A skilled operator could complete the entire operation in about 2 minutes.

## 5. Conclusion

Sandblasting significantly reduced the shear bond strength of refurbished brackets and damaged the microcrystalline structure of the brackets, indicating that this technique is unsuitable for processing ceramic brackets. Although flaming-processed brackets had a bond strength that was similar to that of new ceramic brackets, flaming affected the appearance of the brackets. Exposure to an Er:YAG laser resulted in the complete removal of adhesive from the base of ceramic brackets without damaging them and the shear bond strength of recycled brackets was similar to that of new brackets. In hospitals and private clinics where an Er:YAG laser is available and applied in oral medicine or dental surgery, orthodontist can also use it to refurbish the dislodged ceramic brackets and rebond it to the same patient, which is more effective than traditional methods.

## Figures and Tables

**Table 1:** Comparison of shear bond strength (N).

Group	*n*	Mean ± standard deviation	*p*
New	15	286.053 ± 82.857	—
Flame	15	278.894 ± 56.201	0.79
Sandblasting	15	117.006 ± 68.049	0.00^*∗*^
Er:YAG laser	15	282.590 ± 77.953	0.90

^*∗*^ *p* < 0.05 versus the control group.

**Table 2:** ARI scores.

Group	*n*	0	1	2	3	Total
New	15	4	2	1	8	28
Flame	15	2	2	6	5	29
Sandblasting	15	0	1	3	11	40
Er:YAG laser	15	2	3	5	5	28
